# Under (stromal redox) pressure: NADP^+^ synthesis regulates photosystem I biogenesis

**DOI:** 10.1093/plphys/kiac235

**Published:** 2022-06-28

**Authors:** Gustaf E Degen

**Affiliations:** School of Biosciences, University of Sheffield, Sheffield S10 2TN, UK

Virtually all life on Earth depends on photosynthesis, a process that converts light energy into chemical energy and assimilates CO_2_ into sugars and organic compounds that form the basis of food and fuel. Plants use photosystems I and II (PSI/II) to transfer electrons along the linear electron transfer (LET) chain to NADP^+^ and to generate a proton gradient to drive ATP synthesis. Electrons are extracted from water at PSII and are transferred via several electron carriers to plastocyanin (PC). Electrons are then transferred from PC through PSI to NADP^+^ to produce NADPH. The reducing power stored in NADPH is used for carbon fixation and nitrogen assimilation and is crucial for maintaining redox homeostasis in chloroplasts. In addition to LET, PSI also uses a cyclic pathway (cyclic electron transfer [CET]) to produce ATP without generating NADPH. The CET-mediated increased ATP/NADPH ratio protects PSI from damage caused by stromal overreduction. Hence, proper partitioning between LET and CET is important to maintain PSI function and stability. PSI assembly, structure, and function are well-characterized and biogenesis is affected by the stromal redox state (Heinnickel et al., 2016; Zhu et al., 2016). However, underlying regulatory mechanisms that modulate assembly are not yet well characterized.

In this issue of *Plant Physiology*, Ji et al. (2022) uncovered a regulatory mechanism between PSI biogenesis and stromal redox balance. They demonstrated that PSI biogenesis is downregulated when NAD kinase (NADK) 2, which catalyses the biosynthesis of NADP^+^ from NAD^+^, is compromised.

NADKs are present in all living organisms investigated to date. NADP^+^ synthesis is crucial in chloroplasts since it is the final electron acceptor of the electron transfer chain. Arabidopsis (*Arabidopsis thaliana*) has three NADKs (NADK1–3). NADK2 is chloroplast-localized and plants without NADK2 display stunted growth, increased sensitivity to environmental stresses, impaired chlorophyll synthesis, and reduced photosynthetic efficiency (Chai et al., 2005; Takahashi et al., 2006). Overexpressing *NADK2*, on the other hand, leads to increased Rubisco activity, increased nitrogen assimilation, and altered gene expression associated with nitrate metabolism. However, the overall redox state remains unaffected by higher levels of NADK2 (Takahashi et al., 2009). In a similar study in rice (*Oryza sativa*), overexpression of *NADK2* increased photosynthetic efficiency and tolerance of oxidative damage (Takahara et al., 2010).

Ji et al. were interested in identifying additional components involved in chloroplast biogenesis and screened Arabidopsis T-DNA insertion lines for mutants with decreased chlorophyll content, identifying a line with a mutation in the *NADK2* gene. The *nadk2* mutant was less efficient in photosynthesis and had more nonphotochemical chlorophyll fluorescence quenching (NPQ), which measures dissipation of absorbed light energy into heat. More importantly, the authors revealed that the plastoquinone (PQ) pool was more reduced in *nadk2*. Since PQ is upstream of PC and PSI, these findings indicate that electron transfer from PQ to PSI was impaired.

Therefore, the authors investigated PSI activity by measuring P700 oxidation using far red light, revealing that *nadk2* had much less photo-oxidizable PSI. Furthermore, P700 oxidation was much slower in *nadk2* than in WT, suggesting that the PQ pool was either re-reduced much faster or PSI was limited by downstream electron acceptors, such as NADP^+^, which would be consistent with the roles of NADK2 in NADP^+^ biosynthesis, resulting in disrupted redox homeostasis in the chloroplast stroma. The increased acceptor-side limitation of PSI in *nadk2* prompted the authors to investigate rates of CET, which can act as an electron sink. Indeed, CET around PSI was increased. Furthermore, the *nadk2* mutant was much more sensitive to photoinhibition, due to the diminished accumulation of PSI subunits. The PSI core complex consists of 15 subunits, with PsaA and PsaB proteins forming the central heterodimer holding the reaction center P700 and further components of the electron transfer chain (Amunts et al., 2010). PSI assembly starts by insertion of PsaA and PsaB into the thylakoid membrane and the PsaAB heterodimer reaction center is formed after binding of co-factors, followed by attachment of small subunits (Schöttler et al., 2011). Interestingly, the decreased accumulation of PsaA and B was not caused by more protein turnover in *nadk2*. Instead, the authors revealed translation of *psaA/B* mRNA was downregulated in *nadk2*, providing a link between NADP^+^ levels mediated by NADK2 and PSI biogenesis (Figure 1).

**Figure 1:**
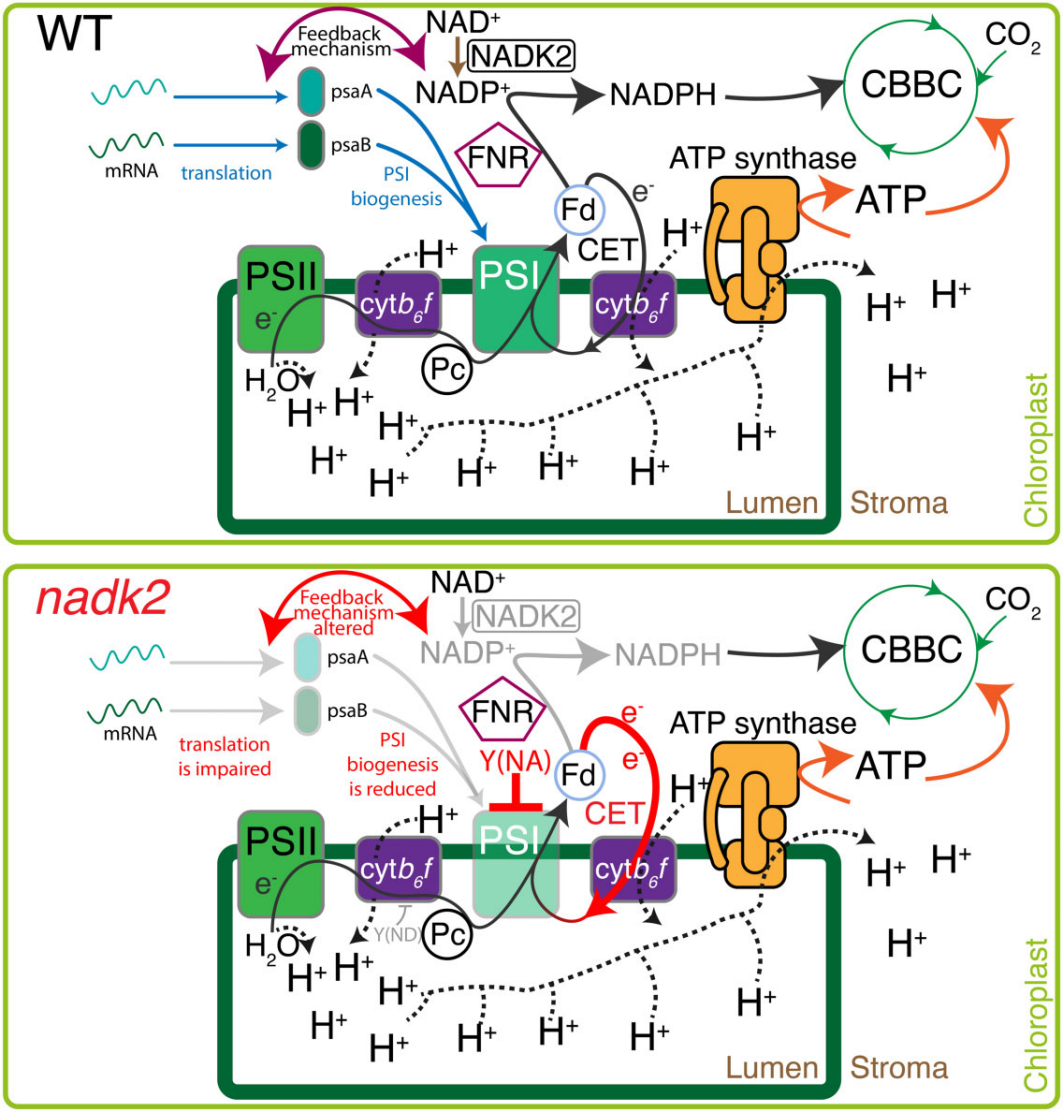
Summary of regulatory crosstalks between NADP^+^ synthesis and PSI biogenesis. NADP^+^ synthesis in WT is catalyzed by NADK2, resulting in normal stromal redox pressure and, as a result, WT-level *PsaA/B* translation and PSI biogenesis. In *nadk2*, NADP^+^ synthesis is disturbed, leading to altered redox pressure in the stroma. This results in downregulation of *PsaA/B* translation and PSI biogenesis to protect PSI from photoinhibition, leading to increased acceptor-side limitation and cyclic electron transfer around PSI (CET) (Ji *et al.*, 2022). Solid arrows represent electron flow. Dotted arrows indicate proton flow. Changes in *nadk2* are highlighted. cyt*b_6_f*, cytochrome *b_6_f* complex; Fd, ferredoxin; FNR, Fd:NADP(H) oxidoreductase, CBBC, Calvin–Benson–Bassham cycle; Y(ND), donor-side limitation; and Y(NA), acceptor-side limitation.

As plants experience environmental changes, such as varying light intensities and temperature, chloroplast metabolism must respond accordingly. The regulatory link between NADP^+^ synthesis, modulated by NADK2, and PSI biogenesis uncovered by Ji et al. provides a mechanism by which plants can adjust PSI levels in response to stromal redox pressure.

*Conflict of interest statement*. None declared.

## References

[kiac235-B1] Amunts A , Toporik H, Borovikova A, Nelson N (2010) Structure determination and improved model of plant photosystem I. J Biol Chem 285: 3478–3486 19923216 10.1074/jbc.M109.072645 PMC2823434

[kiac235-B2] Chai M-F , Chen Q-J, An R, Chen Y-M, Chen J, Wang X-C (2005) NADK2, an Arabidopsis chloroplastic NAD kinase, plays a vital role in both chlorophyll synthesis and chloroplast protection. Plant Mol Biol 59: 553–564 16244906 10.1007/s11103-005-6802-y

[kiac235-B3] Heinnickel M , Kim RG, Wittkopp TM, Yang W, Walters KA, Herbert SK, Grossman AR (2016) Tetratricopeptide repeat protein protects photosystem I from oxidative disruption during assembly. Proc Natl Acad Sci USA 113: 2774–2779 26903622 10.1073/pnas.1524040113 PMC4791029

[kiac235-B2286297] Ji D, , Li Q,, Guo Y,, An W,, Manavski N,, Meurer J,, Chi W (2022) NADP+ supply adjusts the synthesis of photosystem I in Arabidopsis chloroplasts. Plant Physiol **189**: 2128–2143 10.1093/plphys/kiac161 PMC9343004 35385122

[kiac235-B4] Schöttler MA , Albus CA, Bock R (2011) Photosystem I: Its biogenesis and function in higher plants. J Plant Physiol 168: 1452–1461 21255865 10.1016/j.jplph.2010.12.009

[kiac235-B5] Takahara K , Kasajima I, Takahashi H, Hashida S, Itami T, Onodera H, Toki S, Yanagisawa S, Kawai-Yamada M, Uchimiya H (2010) Metabolome and photochemical analysis of rice plants overexpressing Arabidopsis NAD kinase gene. Plant Physiol 152: 1863–1873 20154096 10.1104/pp.110.153098 PMC2850022

[kiac235-B6] Takahashi H , Watanabe A, Tanaka A, Hashida S, Kawai-Yamada M, Sonoike K, Uchimiya H (2006) Chloroplast NAD kinase is essential for energy transduction through the xanthophyll cycle in photosynthesis. Plant Cell Physiol 47: 1678–1682 17082216 10.1093/pcp/pcl029

[kiac235-B7] Takahashi H , Takahara K, Hashida S, Hirabayashi T, Fujimori T, Kawai-Yamada M, Yamaya T, Yanagisawa S, Uchimiya H (2009) Pleiotropic modulation of carbon and nitrogen metabolism in Arabidopsis plants overexpressing the NAD kinase2 gene. Plant Physiol 151: 100–113 19587098 10.1104/pp.109.140665 PMC2735975

[kiac235-B8] Zhu Y , Liberton M, Pakrasi HB (2016) A novel redoxin in the thylakoid membrane regulates the titer of photosystem I. J Biol Chem 291: 18689–18699 27382055 10.1074/jbc.M116.721175 PMC5009245

